# Genomic analysis of expressed sequence tags in American black bear *Ursus americanus*

**DOI:** 10.1186/1471-2164-11-201

**Published:** 2010-03-26

**Authors:** Sen Zhao, Chunxuan Shao, Anna V Goropashnaya, Nathan C Stewart, Yichi Xu, Øivind Tøien, Brian M Barnes, Vadim B Fedorov, Jun Yan

**Affiliations:** 1 CAS-MPG Partner Institute for Computational Biology, Shanghai Institutes of Biological Sciences, 320 Yue Yang Road, Shanghai, 200031, China; 2 Institute of Arctic Biology, University of Alaska Fairbanks, Fairbanks, AK, 99775, USA; 3 Current address: Microbial Evolution Research Group (MERG), Department of Biology, University of Oslo, N-0316 Oslo, Norway

## Abstract

**Background:**

Species of the bear family (*Ursidae*) are important organisms for research in molecular evolution, comparative physiology and conservation biology, but relatively little genetic sequence information is available for this group. Here we report the development and analyses of the first large scale Expressed Sequence Tag (EST) resource for the American black bear (*Ursus americanus*).

**Results:**

Comprehensive analyses of molecular functions, alternative splicing, and tissue-specific expression of 38,757 black bear EST sequences were conducted using the dog genome as a reference. We identified 18 genes, involved in functions such as lipid catabolism, cell cycle, and vesicle-mediated transport, that are showing rapid evolution in the bear lineage Three genes, Phospholamban (*PLN*), cysteine glycine-rich protein 3 (*CSRP3*) and Troponin I type 3 (*TNNI3*), are related to heart contraction, and defects in these genes in humans lead to heart disease. Two genes, biphenyl hydrolase-like (*BPHL*) and *CSRP3*, contain positively selected sites in bear. Global analysis of evolution rates of hibernation-related genes in bear showed that they are largely conserved and slowly evolving genes, rather than novel and fast-evolving genes.

**Conclusion:**

We provide a genomic resource for an important mammalian organism and our study sheds new light on the possible functions and evolution of bear genes.

## Background

Studies of molecular evolution and the genetic basis of physiological adaptations to extreme environments are limited by the lack of comparative genomic resources including non-model species. Species of the bear family (*Ursidae*) are interesting and important organisms for research in molecular evolution, comparative physiology and conservation biology, but relatively little genetic sequence information is available among species. A major family in the order Carnivora, it consists of eight extant species: giant panda, spectacled bear, and six species within the *Ursus *genus (brown, polar, sloth, sun, and Asiatic and American black bears). The phylogeny of the bear family has been intensively studied using mitochondrial genomes [1-3], selected nuclear gene markers [4], or fragmented nuclear DNA sequences [5]. However currently, there are only 2,565 nucleotide sequences from the *Ursidae *family in the NCBI database, mostly corresponding to mitochondrial genes. The genome sequence of the giant panda (*Ailuropoda melanoleuca*), a species that split off from the *Ursus *bear genus about 12 million years ago (MYA) [2], was released after the completion of this study [6]. The availability of large-scale genomic resources for a bear species should facilitate the study of molecular evolution in the bear family and development of population genetic markers to address conservation issues.

Bears are of interest for the study of the molecular and genetic basis of mammalian hibernation. Brown bear (*U. arctos*) and black bear (*U. americanus*) of both sexes hibernate, and females of polar bears (*U. maritimus*), hibernate when pregnant [7]. Hibernation is an energy saving adaptation that is utilized by at least seven orders of mammals to survive in unpredictable or seasonally extreme environments [8]. Entry into hibernation is signified by profound reduction in whole animal metabolism (to 2-25% basal rates), regulated decreases in body temperature, heart beat and metabolic rates that persist over hibernation seasons that can span nearly eight months. Most hibernation studies conducted so far have focused on the hibernators of small body sized such as ground squirrels (<1 kg) [9-12] and marmots (<10 kg) [13,14]. However, bears (50-250 kg) demonstrate a unique pattern of winter hibernation as they remain at relatively high body temperatures and, although they keep quiet and largely immobile, they are capable of arousing and moving throughout the four-eight month hibernation period. During hibernation, bears do not eat, urinate, or defecate [15]. They hibernate with a 20-50% reduction in metabolic rate and the reduction of heart rate from 60 bpm to 10 bpm [16,17]. Although they are largely inactive during hibernation, they show no loss in bone mass and less loss in muscle mass and function than is anticipated over such a prolonged state of immobilization [18]. In contrast to the near-freezing body temperature in small-sized hibernators ([19], core body temperatures of black bears (30-150 kg) only decrease to 30-36°C. The periodic temperature cycling in hibernating bears is also much less than in the torpor-arousal cycle of ground squirrels. Our understanding of the molecular mechanisms that regulate bear hibernation could lead to creating novel therapies for treating human conditions related to resistance to trauma and recovery during rehabilitation.

To develop genomic resources for the American black bear (*Ursus americanus*), the most common and widely distributed bear species in North America, we have constructed cDNA libraries and sequenced nearly 40,000 Expressed Sequence Tag (EST). An initial study utilizing these ESTs to construct cDNA arrays and detect gene expression changes during bear hibernation has been reported elsewhere [20]. In this study, we focus on the in-depth analyses of the bear ESTs collection to obtain the first insights into biological functions, alternative splicing, tissue-specificity of expression, and molecular evolution of genes in the bear genome.

## Results and Discussion

### Bear EST sequencing and alignments on the dog genome

A total of 38,757 EST sequences from *Ursus americanus *were generated from cDNA libraries in brain, liver, heart, skeletal muscle, and testis (Table 1) using a normalization-subtraction method (See *Methods*). EST lengths ranged from 13 - 1,042 base pairs (bps), average 500 bps (Additional file 1, Figure S1; NCBI dbEST database [21] with accession numbers: GW276093 - GW314849).

**Table 1:** Statistics of EST numbers from different bear tissues.

Tissue	Total ESTs
Liver	11,191
Brain	11,300
Skeletal Muscle	6,010
Heart	8,297
Testis	1,959

To identify the relative genomic positions and the splicing patterns of bear ESTs, we mapped these 38,757 ESTs onto the dog genome by using the procedure described in [22]. Dog (*Canis familiaris*) separated from bears by about 59.2 MYA [23]. Bear ESTs were first masked by RepeatMasker [24] and aligned on the dog genome, downloaded from ENSEMBL, by BLASTN [25]. SIM4 [26] was used to identify splicing sites in corresponding BLAST-hit segments (See *Methods*). 32,561 (84.0%) bear ESTs can be mapped on the dog genome. The distribution of the percentage of identities between the aligned bear ESTs and dog genome is shown in Figure S2 (Additional file 2, Figure S2). On average, bear EST sequences demonstrated a 91.0% sequence identity with dog.

We further clustered the aligned bear ESTs with the help of annotated dog mRNA transcripts. The clustering process was based on the shared splice sites or the length of overlap, i.e. two sequences were clustered together if they shared at least one splice site at the same orientation, or if they overlap more than half of the length of the shorter sequence on the genome. After clustering, we obtained 18,297 "primary EST clusters". The redundant "primary EST clusters" that aligned to multiple genomic loci were further merged together. Finally, we obtained 10,644 "unique bear EST clusters". We selected the primary cluster with the most ESTs to represent "unique bear EST clusters". Most of the unique bear EST clusters (6,409 clusters, 60.2%) are singletons, i.e. containing only one EST. The average number of ESTs per unique bear EST cluster is 3.07. The distribution of numbers of ESTs per unique EST cluster is shown in Figure S3 (Additional file 3, Figure S3). The low EST copy number per EST cluster and high numbers of unique EST clusters indicate that normalization subtraction method used in cDNA library construction has substantially increased transcript diversity in our EST project.

The remaining 6,196 ESTs that failed to be mapped onto dog genome were assembled into 4,846 EST clusters by CAP3 [27]. Most (4,352 clusters, 90%) of these EST clusters were singletons. The average number of ESTs per cluster is 1.28. In contrast to the EST clusters mapped on dog genome, these clusters mostly represent rare transcripts.

### EST Annotation

All bear ESTs were aligned against human RefSeq mRNAs http://www.ncbi.nlm.nih.gov/ by BLASTN. A BLAST score higher than 100 was used as the criterion of homologous match. Annotation revealed that 29,160 (75.2%) ESTs matched human gene symbols. A total of 14,984 (82%) "primary EST clusters" corresponding to 7,680 (72%) "unique bear EST clusters" were annotated with human gene symbols. For the remaining "unique bear clusters", the longest EST in each cluster was aligned against NCBI NT database, which contains a much boarder collection of nucleotide sequences. Using alignment score higher than 100 as the cutoff, we annotated 778 "unique bear EST clusters" with known gene symbols. Among the 1,490 "unique bear EST clusters" without annotation, only nine contained more than five ESTs and seven had an alignment score higher than 50 when aligned with NT sequences. This suggested that they were mostly rare unknown transcripts. In total, we obtained 8,458 (79%) "unique bear EST clusters" with known gene symbols.

For the ESTs that did not align onto the dog genome, the contig sequences generated by CAP3 were aligned against the NT database with BLASTN and a cutoff of score higher than 100 was used again. A total of 1,231 (25%) EST clusters were annotated with known gene symbols.

**Figure 1:**
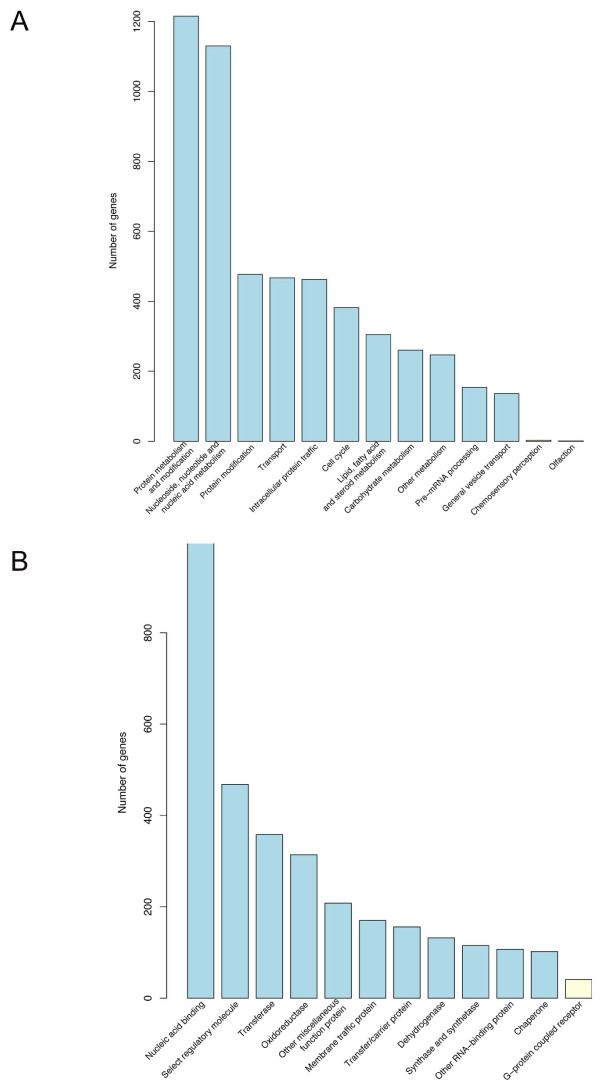
**Enrichment analysis of GO annotations in bear genes**. (A) Genes that were over- or under-represented in biological processes terms. (B) Genes that were over- or under-represented in molecular function terms. Both biological processes and molecular functions were selected with the criteria of P-value ≤ 1.0 × 10^-10^. Blue bar represents the over-represented process and yellow bar represents the under-represented process.

A total of 7,986 EST non-redundant clusters from black bear were annotated with known gene symbols and submitted to PANTHER [28] for functional annotations. A total of 4,471, 4,558, and 1,013 genes were classified according to biological process, molecular function, and biological pathway, respectively. Genes involved in 11 categories of biological processes were significantly over-represented (P-value < 1.0 × 10^-10^, Bonferroni-corrected) in the EST collection (Figure 1A) [29]. "Protein metabolism and modification" and "Intracellular protein traffic" categories were the most significantly over-represented in terms of biological processes. Genes involved in the two categories, "olfaction" and "chemosensory perception", of biological processes were significantly under-represented. Genes assigned to 11 categories of molecular functions were significantly over-represented in the EST collection. The "oxidoreductase" category was the most significantly over-represented molecular function, whereas "G-protein coupled receptor" category was significantly under-represented (Figure 1B). There was no biological pathway category that passed the criterion (P-value < 1.0 × 10^-10^) to be significantly enriched.

### Tissue-specific bear gene expression

**Figure 2:**
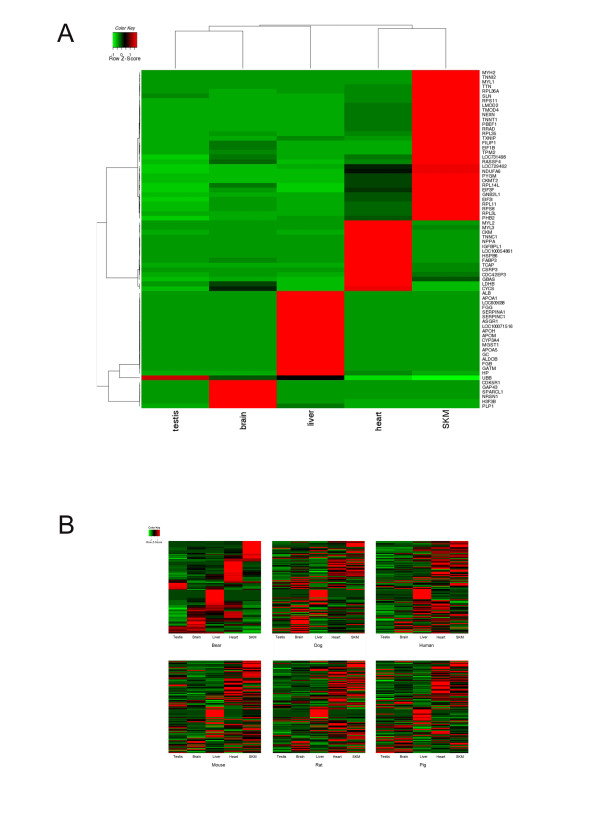
**Heatmap representation of gene expression of tissue specific genes across liver, brain, heart, testis, and skeletal muscle (SKM)**. (A) 72 bear tissue-specific genes; (B) Comparison of tissue-specific expression of 108 genes across six species: bear, human, mouse, rat, dog, and pig. The order of the genes was arranged according to the clustering of gene expression in bear.

We used EST copy numbers from brain, liver, heart, skeletal muscle, and testis as an approximate estimate of gene expression level across these five tissues [30]. We performed Fisher's exact test on 527 clusters with at least 10 ESTs and identified 72 tissue specific clusters under the criteria (P-value < 10^-3^, odds ratio > 10). Among them, six, 18, 15, 32, and one clusters are brain, liver, heart, skeletal muscle, and testis-specific, respectively. Their expression levels estimated by EST copy numbers are shown in Figure 2A. Myosin heavy chain 2 (*Myh2*) and troponin I type 2 (*Tnni2*), involved in muscle contraction, were significantly over-represented in skeleton muscle. Albumin (*Alb*), involved in fatty acid transport, was significantly over-represented in liver. Growth associated protein 43 (*Gap43*), associated with neuronal growth cones, demonstrated highly brain-specific expression. Myosin light chain 2 (*Myl2*), associated with cardiac myosin beta chain, was over-represented in heart.

To compare our results with tissue specific gene expression in other mammals, we downloaded gene expression profiles estimated from EST copy numbers of human (*Homo sapiens*), mouse (*Mus musculus*), rat (*Rattus norvegicus*), dog (*Canis lupus familiaris*) and pig (*Sus scrofa*) from the NCBI Unigene database http://www.ncbi.nlm.nih.gov/unigene. Data on expression of 108 homologous genes were obtained in all six species across the same five tissues (Figure 2B, see *Methods*). We first calculated the Pearson correlation coefficients (r) in pair-wise comparisons of gene expressions as estimated from EST copy numbers between species for each tissue. The gene expression is highly conserved in liver across species with average correlation ⟨*r*⟩ = 0.87, while the correlations are much lower in the other four tissues with ⟨*r*⟩ as 0.44, 0.41, 0.45, 0.30 for heart, skeletal muscle, brain, and testis respectively. We also calculated the correlation coefficients in pair-wise comparisons between tissue gene expressions among species for each gene. A total of 54 genes had consistent expression profiles between bear and at least one out of five other species (Pearson correlation r > 0 and P-value < 0.05). Only 16 out of 108 genes are highly consistent in tissue specific expression profile between any two species among all six species. For example, apolipoprotein A-I (*Apoa1*), a major component of high density lipoprotein, was significantly over-represented in liver in all six mammals. Proteolipid protein 1 (*Plp1*), encoding the most abundant myelin protein in the central nervous system, was highly expressed in brain in all six mammals. However, most of other studied genes demonstrated species-specific patterns of expression across tissues.

### Analysis of splicing variants

**Figure 3:**
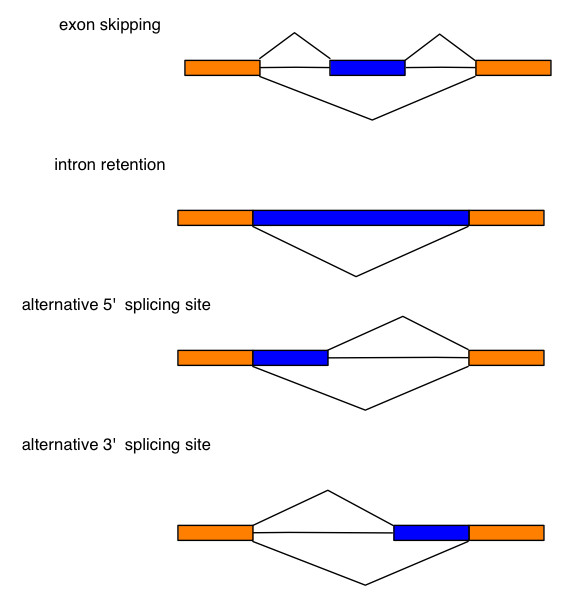
**Four types of alternative splicing patterns: exon skipping, intron retention, alternative 5' splicing site, and alternative 3' splicing site**.

Alternative splicing is an important mechanism that generates transcript diversity. It is estimated that 94% of genes may undergo alternative splicing in humans [31]. The alignment of bear ESTs on dog genome provided the opportunity to reveal alternative splicing patterns in bear. After removing all ambiguously spliced and non-spliced bear ESTs, we obtained 2,512 unique bear EST clusters with at least two spliced bear ESTs containing 15,458 bear ESTs for alternative splicing analysis. A total of 630 clusters (25%) showed alternative splicing and 504 alternative splicing events were classified into four typical alternative splicing patterns [32]: alternative 5' site (159 events), alternative 3' site (141 events), exon skipping (145 events), and intron retention (59 events) (Figure 3).

To estimate the proportion of conserved splicing events between bear and dog, we compared the spliced patterns in bear with those in annotated dog mRNA transcripts. There are 4,311 unique bear EST clusters containing at least one spliced bear EST and at least one spliced dog mRNA transcript. Among 37,874 splice sites in these clusters, 32,567 (86%) of them can be also found in dog mRNA transcripts and 2,225 (52%) of 4,311 unique bear EST clusters contain splice sites completely identical to those in dog mRNA transcripts.

Kim *et al*. [32] reported that exon skipping is the most abundant alternative splicing events in human, mouse and rat, and that intron retention is the rarest event. To compare our results to alternative splicing pattern in other mammals, we normalized our EST database as described in Kim et al. [32] by randomly selecting the same number of ESTs from the EST collection (see Methods). We observed that about 42% unique bear EST clusters were alternatively spliced, consistent with previous findings in other mammals [32]. Our result revealed that alternative 5'site had the highest occurrence (35%), followed by alternative 3'site (29%), exon skipping (24%), and intron retention (12%). The proportions of four typical alternative splicing patterns in four mammalian species are shown in Table 2. Frequency of alternative splicing patterns in bear was not significantly different from distribution of splicing events in other mammalian species (P = 0.3, Chi square test).

**Table 2:** Comparison of alternative splicing patterns in four species.

Species	Exon skipping (%)	Alternative 3' splice site (%)	Alternative 5' splice site (%)	Intron retention (%)
Human	42	26	24	8
Mouse	37	28	26	9
Rat	36	28	31	5
Bear	24	29	35	12

The percentages of four alternative splicing patterns in human, mouse and rat were extracted from Eddo kim's paper [32]. The result for bear was obtained after the normalization.

### EST coverage of protein-coding regions

**Figure 4:**
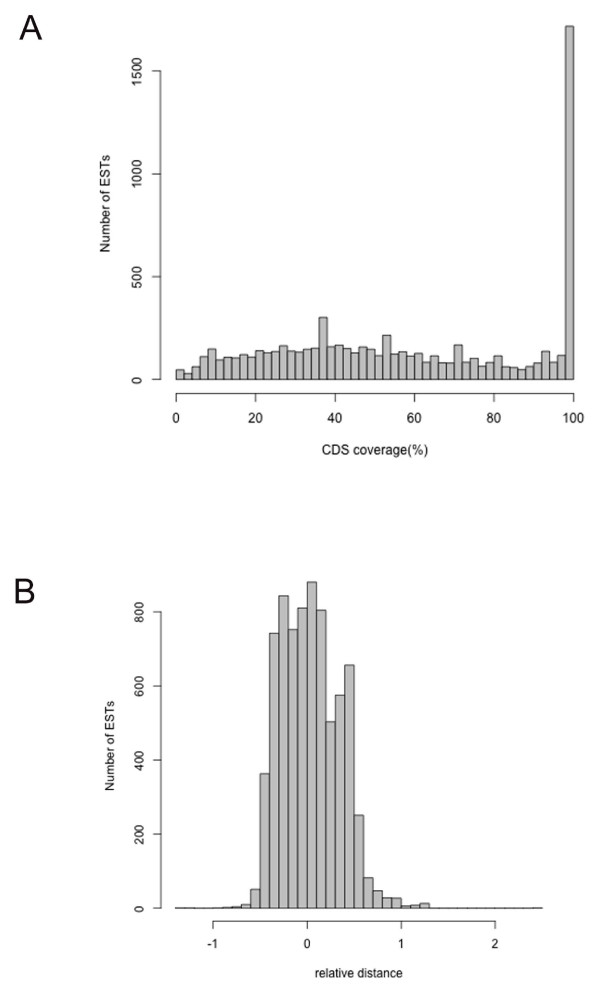
**The distribution of the bear ESTs on CDS regions**. (A) Distribution of CDS coverage among bear ESTs. About 1,600 bear ESTs contained full length CDS region. (B) Distribution of bear EST positions relative to the center of CDS region.

To estimate the coverage of protein-coding regions in the bear EST collection, we selected spliced bear ESTs with identical splicing sites with spliced dog transcripts or unspliced ESTs overlapped with unspliced dog transcripts. We discarded dog transcripts with ambiguous start and stop codons. Bear ESTs aligned on more than one gene or completely fell into the non-coding regions were also discarded. Finally, we obtained 7,461 bear ESTs and calculated the positions of 5' and 3' ends of bear ESTs on dog transcripts. For these ESTs, the average coverage of protein-coding regions was 59.2%. In total, 1,619 ESTs contained complete protein coding regions, corresponding to 395 ENSEMBL-annotated dog genes. The distribution of the coverage of protein coding regions was shown in Figure 4A. There is no obvious bias towards 5' or 3' end of protein-coding regions in bear ESTs (Figure 4B).

### Molecular evolution in bear

The phylogenetic relationships between bear and other mammalian species have been previously inferred from mitochondrial sequences and a few selected nuclear gene markers [2,3]. Thus, we had the opportunity to compare the phylogenetic results derived from the bear mitochondrial genes with a phylogeny from a large collection of nuclear genes. A total of 15,304 bear ESTs with coding regions were further assembled into 5,356 EST contigs by CAP3. The protein-coding regions accounted for 72% of all assembled contig sequences. The high-quality genome assemblies from three other mammalian species: cow, dog, and human were downloaded from ENSEMBL. Orthologous gene annotations were obtained from Ensembl-Compara database. Multiple sequence alignments of orthologous genes were generated by MUSCLE program.

Maximum likelihood method was used to reconstruct the phylogenetic tree of the four species from a concatenated alignment of the coding regions of 2,655 nuclear genes and 13 mitochondrial genes respectively with the GTR nucleotide substitution model after multiple hit corrections (Additional file 4, Figure S4). The phylogenetic tree derived from the bear nuclear genes has the same topology as the one derived from mitochondrial genes. Then, Ka (non-synonymous substitution rate) and Ks (synonymous substitution rate) values of each lineage were calculated under the free ratio model in PAML package (Table 3). Although there are considerable variations of Ka and Ks among different mammalian lineages as estimated from nuclear genes as well as mitochondrial genes, the ratios between Ka and Ks (Ka/Ks) from nuclear genes showed smaller variation among species compared to mitochondrial genes. Ka/Ks in the bear lineage estimated from nuclear genes was compatible with those in other lineages (Table 3), indicating that the selection constraint in bear did not show significant differences from other mammals on the global nuclear genomic scale.

**Table 3:** The evolutionary rates along each lineage in the phylogenetic tree of four mammalian species.

	Nuclear			Mitochondria		
	Ka	Ks	Ka/Ks	Ka	Ks	Ka/Ks
Bear	0.0102	0.0798	0.1278	0.0365	1.0037	0.0363
Dog	0.0092	0.0843	0.1091	0.0274	1.2123	0.0226
Cow	0.0189	0.1657	0.1143	0.0343	1.4843	0.0231
Human	0.0186	0.1517	0.1226	0.1266	2.2890	0.0553

Rates of evolution were estimated from the alignment of concatenated nuclear and mitochondria coding sequences; (Ka) nonsynonymous substitution per codon; (Ks) synonymous substitution per codon.

Next, we searched for specific genes with rapid evolutionary rates in the bear lineage using the likelihood ratio test implemented by PAML program. In total, 154 genes had significantly higher Ka/Ks values (P < 0.05, likelihood ratio test (LRT)) in the bear lineage than in the other lineages (Additional file 5, Table S1). After manually checking the alignments, the results of 18 genes with coding sequence (CDS) coverage larger than 80% were listed together with their biological functions in Table 4. They are involved in diverse biological functions including PNPLA4 in lipid catabolism, CDC42SE1 in cell cycle, and TMED2 in vesicle-mediated transport. In particular, three genes: Phospholamban (*PLN*), cysteine glycine-rich protein 3 (*CSRP3*), and Troponin I type 3 (*TNNI3*), are involved in heart contraction. *PLN *inhibits cardiac muscle sarcoplasmic reticulum Ca^2+ ^pump when it is unphosphorylated. Upon *PLN*'s phosphorylation, Ca^2+ ^pump will be activated leading to muscle relaxation. Defects in *PLN *in human are a cause of dilated cardiomyopathy 1P (CMD1P). CSRP3 is expressed specifically in heart (Figure 2A). CSRP3 is associated with regulation of heart contraction and skeletal muscle development. Defects in CSRP3 are a cause of dilated cardiomyopathy 1M (CMD1M). *TNNI3 *is the cardiac protein of troponin I, the inhibitory subunit of troponin. Troponin I blocks actin-myosin interactions and plays an important role in the relaxation of striated muscle. Defects in this gene in human can lead to familial hypertrophic cardiomyophathy type 7 (CMH7) and restrictive cardiomyopathy (RCM).

**Table 4:** The bear genes showing rapid evolution in bear lineage (CDS coverage ≥ 80%).

Gene Symbol	**P value**^**a**^			**CDS Coverage **^**d**^	**Biological functions**^**e**^
PNPLA4	0.001	0.145	0.649	0.984	Lipid catabolic process
CDC42SE1	0.001	0.0001	0.433	0.987	Signal transduction; Regulation of cell shape
CNPY2	0.002	0.027	0.402	1	Unknown
TMED2	0.011	0.006	0.211	1	Vesicle-mediated transport;
CSRP3	0.012	0.014	0.113	0.994	Regulation of the force of heart contraction; Cellular calcium ion homeostasis;
CHCHD1	0.016	0.144	4.156	0.991	Unknown
TNNI3	0.017	0.018	0.145	0.86	Cardiac muscle contraction; Regulation of systemic arterial blood pressure by ischemic conditions; Cellular calcium ion homeostasis;
APOH^L^	0.018	0.226	0.506	0.962	Negative regulation of endothelial cell proliferation; Triacylglycerol metabolic process; Blood coagulation, intrinsic pathway;
C1orf52	0.019	0.078	0.711	1	Unknown
PLN^M^	0.02	0.058	Inf*	0.981	Regulation of the force of heart contraction; Cellular calcium ion homeostasis; Blood circulation
C19orf39	0.021	0.301	0.948	0.925	Unknown
BPHL	0.024	0.127	0.314	0.859	Proteolysis; Response to toxin; Cellular amino acid and derivative metabolic process
YIPF3	0.028	0.021	0.08	0.854	Cell differentiation
DR1	0.029	0.001	0.191	0.824	Negative regulation of transcription
RBM8A^M^	0.033	0.0001	0.068	0.821	RNA processing; mRNA binding
POLR2C	0.039	0.003	0.037	0.876	Transcription initiation
ATP6V1F^H^	0.042	0.009	Inf*	0.991	Ion transport; ATP synthesis coupled proton transport;
CISD2	0.043	0.011	0.15	1	Regulation of cellular respiration

^a^p-value calculated from the LRT under chi-squared distribution with df = 1.

^b ^ω_1 _is the Ka/Ks value of the bear lineage; ^c ^ω_0 _is the average Ka/Ks value in the rest of lineages.

^d^The coverage of protein coding region of bear EST using human RefSeq sequence as reference.

^e^Biological functions obtained from GO annotations.

* In these cases, the rate of synonymous substitution reaches zero (Ks = 0) and Ka/Ks diverges and represented as Inf here.

^H, L, M^The gene shows significantly differential expression (P < 0.05, one-way ANOVA) in heart, liver, or skeletal muscle tissue respectively.

**Figure 5:**
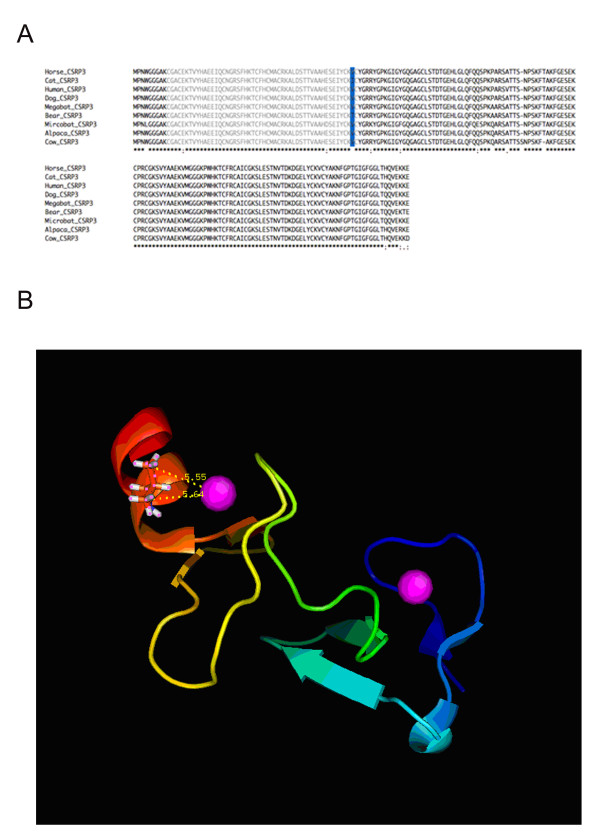
**Protein sequence alignment and 3D protein structure of CSRP3**. (A) Multiple sequence alignment of full-length CSRP3 protein sequences in nine species. The LIM1 zinc-binding domain is marked with grey and the 60^th ^site is highlighted in blue. The corresponding nucleotide sequence alignment is shown in Figure S5 (Additional file 6, Figure S5). (B) 3D protein structure of human CSRP3 LIM1 domain obtained from Protein Database (PDB id: 2o13). Two dash lines show distances between the zinc ion and the methyl groups of the valine residue.

We then used branch-site model to test whether any codon sites of the rapid evolving genes show positive selection signal in the bear lineage. Two of 18 genes, biphenyl hydrolase-like (*BPHL*) and cysteine glycine-rich protein 3 (*CSRP3*), contained codon sites with the Ka/Ks significantly greater than one (P < 0.05) as estimated by the Bayes empirical Bayes (BEB) method [33]. We subsequently added protein coding sequences of *BPHL *and *CSRP3 *from more mammalian species including cat, mircobat, megabat, alpaca, horse and hedgehog, to the four species and tested whether this would affect the results of positive selection detection. The candidate site (19I) in *BPHL *still showed 99.1% probability of being positively selected. *BPHL *has hydrolase activity and may be involved in detoxification process. The putative positive selected site (19I) in *BPHL *fell in the signal peptide domain. For *CSRP3*, although the bear lineage still showed a higher Ka/Ks value comparing to the rest of the phylogeny (P = 0.02, LRT), the candidate site (60T) was identified now with a lower probability (88.7%) of being positively selected along the bear lineage. The putative positive selected site (60T) in *CSRP3 *fell in the zinc-binding domain. The multiple alignment of *CSRP3 *protein sequences was shown in Figure 5A and corresponding nucleotide sequence alignment shown in Figure S5 (Additional file 6, Figure S5). There was also the substitution of valine by isoleucine at the 60^th ^codon in cat lineage. We obtained 3-D structure of human *CSRP3 *proteins from Protein Data Bank (PDB) database (PDB id: 2o13). The amino acid change from valine (Val) in human, cow, horse, microbat, megabat, alpaca, and dog to threonine (Thr) in bear at 60^th ^amino acid may potentially affect the structure of its N-terminal LIM domain. This is likely to change the conformation of domain by introducing a polar interaction between a hydroxyl group of the threonine and the zinc ion (Figure 5B). In contrast, the substitution by isoleucine, another aromatic acid, at this site in the cat lineage would not change the conformation of LIM1 domain as much as the threonine substitution does in bear lineage. Experiments in human have demonstrated that the altered conformation of *CSRP3 *from the mutations at 44^th^, 55^th ^and 58^th ^site in LIM1 domain can lead to a decreased binding activity of muscle LIM protein (MLP) to α-actinin and titin-cap (T-cap or telethonin) [34]. MLP/T-cap complex is a key component of cardiac mechanical stretch sensor system. The defect in *CSRP3 *may result in human dilated cardiomyopathy and heart failure [35].

### Evolution rate of hibernation-related genes

The phylogenetic distribution of hibernating and non-hibernating species is wide-ranging and interspersed. It is highly unlikely that hibernation phenotype has arisen independently in these hibernating species. So far, there has been no evidence for the creation of novel genes responsible for hibernation phenotype. Therefore, it is more likely that hibernation results from the differential expression of existing genes that have widely and long existed among mammals [8,12]. To address whether bear genes involved in hibernation were evolving faster or slower, we identified hibernation-related genes as those genes that showed significant differential expression on cDNA arrays between bears sampled during hibernation and non-hibernation season (P < 0.05, one-way ANOVA) [20]. Among the 18 genes that we identified as fast-evolving genes in bear, there were four hibernation-related genes: *APOH *(liver), *PLN *(skeletal muscle), *RBM8A *(skeletal muscle), and *ATP6V1F *(heart). *RBM8A *was over-expressed during bear hibernation, while the other three genes were under-expressed.

**Figure 6:**
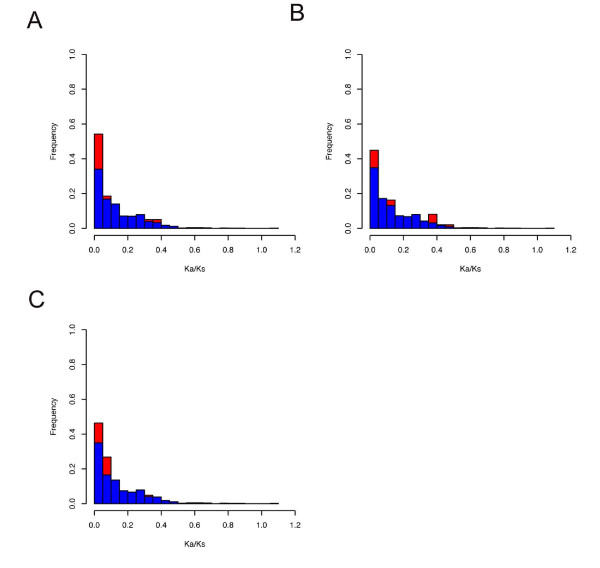
**The distribution of Ka/Ks values (bear vs. dog) for hibernation-related genes (red color) and the rest of genes (blue color) in heart (A), liver (B), and skeletal muscle (C)**.

To further examine the selection pressure on hibernation-related genes on a global scale, we compared the Ka/Ks ratios (bear vs. dog) between hibernation-related genes and other bear genes. The distributions of Ka/Ks values of hibernation-related and other genes are shown in Figure 6. We observed that hibernation-related genes showed a significantly lower Ka/Ks values in heart (P = 5 × 10^-4^, Wilcoxon's rank test) and skeletal muscle (P = 0.01) compared to the rest of the genes, indicating a stronger level of evolutionary constraint on them. This result was also consistent with our previous observation that the proportion of genes involved in protein biosynthesis and translation process was significantly elevated among the over-expressed genes during hibernation [20]. Most of these genes such as ribosomal proteins are housekeeping genes and typically experience highly negative selection [36]. Thus, the genes that control key physiological processes in bear hibernation tend to be more conserved rather than fast evolving.

### Web-based Ursus americanus EST database

A web-based *Ursus americanus *EST database with a user-friendly interface was constructed to provide assess to the bear EST collection and demonstrate our analysis http://www.picb.ac.cn/bearest/bearest.jsp. The queries of bear EST IDs, annotations, tissue sources, and splicing patterns on the dog genome are available on the website.

## Conclusions

In this study, we reported the first collection of 38,757 ESTs of American black bear from normalized subtracted cDNA libraries constructed from five tissues, brain, liver, heart, skeletal muscle, and testis. Assembling these ESTs onto dog genome yielded 10,644 "unique bear EST clusters" indicating that our gene discovery strategy has resulted in wide coverage and low redundancy of the EST collection. Functional annotation and enrichment analyses showed that the EST collection represents genes with diverse biological functions. Some of genes showed significant tissue-specific expression that is comparable with tissue-specific expression of their orthologous genes in other mammalian species. We also studied frequencies of four most common alternative splicing patterns in the bear ESTs sequences [32]. Similar to in other mammals, intron retention is the least common alternative splicing event in the bear. However, alternative 3' or 5' splicing site, instead of exon skipping in other mammals, are the most frequent splicing events in bear.

This first large-scale bear EST project provides a valuable genomic resource to study molecular evolution in bear phylogeny. Using the bear EST collection, we compared the selection pressure exerted on nuclear genes in the bear with those in other mammalian species and identified the genes showing fast evolutionary rate in the bear lineage. These genes have broad biological functions such as metabolism, cell cycle, and vesicle-mediated transport. Three of these genes (*PLN*, *CSRP3*, and *TNNI3*) are associated with cardiac muscle contraction. Two of these genes, *BPHL *and *CSPR3 *contain codon sites showing positive selection signal in the bear lineage. The analysis on the 3D structure of *CSRP3 *zinc-binding domain indicates that this bear-specific amino acid substitution in *CSRP3 *may have a significant impact on its structure. As several heart disease-related mutations have been observed in the same domain in *CSRP3*, it is tempting to suggest that this bear-specific substitution may confer adaptive advantage for bear heart during biological processes such as hibernation. However, molecular evolution leading to adaptation is a complex issue and all fast-evolving and putative positive selected genes identified in this study may or may not be related to hibernation *per se *in bear. The real biological functions and implications related to these genes have yet to be demonstrated. Genes differentially expressed during hibernation showed signs of higher negative selection pressure comparing to other genes. This provides supportive evidence for the hypothesis that the hibernation phenotype results from differential expression of conserved genes, rather than rapid evolutionary origin of novel hibernation specific genes. Future research combining gene expression studies, functional studies, and genome sequencing will shed new light on the evolution of molecular functions in bear species.

## Methods

### Animals

American black bears (31-143 kg) were captured in May-July from the field either near McGrath or Anchorage, Alaska. Bears were transferred to Fairbanks, Alaska, where they were held individually in a shaded outdoor holding facility. After euthanasia, tissue samples were taken in 12 minutes and frozen immediately in liquid nitrogen and stored at -80°C. Animal protocols were approved by the University of Alaska Fairbanks Institutional Animal Care and Use Committee.

### RNA preparation

Total RNA was extracted from frozen tissues by grinding in liquid nitrogen with mortar and pestle and using RNeasy Kit (Qiagen). Skeleton muscle tissue was treated by proteinase K and RNA was extracted by using RNeasy Fibrous Tissue Kit (Qiagen). All RNA samples were processed by DNase I (Qiagen) treatment. For cDNA library construction, mRNA was selected from total RNA with the oligo(dT) cellulose by the use of Poly(A) Purist Kit (Ambion). RNA quality was assessed by 1.2% agarose gel electrophoresis and concentration was measured by using Nanodrop.

### cDNA library construction and sequencing

Normalized cDNA libraries enriched for full-length inserts were constructed from brain, liver, testis, heart, and skeleton muscle. For each library except for testis and liver (hibernating animal only), we pooled mRNA samples isolated from hibernating and summer active bears. During reverse transcription, adaptors containing the rare for mammalian genomes, asymmetrical restriction sites for *SfiI *were incorporated into the first strand of the cDNA using a SMART template switching mechanism at the 5'-end of the transcript [37]. In order to decrease redundancy, most libraries were normalized by hybridization of the single strand cDNA with the same quantity of mRNA that was used for first strand synthesis [38]. To further decrease redundancy, two cDNA libraries from brain (007) and liver (008) were subtracted in addition to normalization. To construct subtraction drivers, 1,000 - 2,000 clones already sequenced from brain (001, 003) and liver (UAhib, 006) libraries were plated on LB-agarose and plasmid DNA was extracted by using QIAprep Spin Miniprep Kit (Qiagen). Using the plasmid DNA as a template, the cDNA inserts were amplified by PCR with M13 universal primers. RNA subtraction drivers were obtained from PCR product by using in vitro transcription with Maxi Script T7 Kit (Ambion). The RNA subtraction driver was pooled with normalization driver, labeled with biotin, and hybridized with the first strand cDNA using the same protocol as used for normalization [38].

Second strand synthesis of cDNA was performed by the use of primer extension PCR (Advantage 2 Taq Polymerase, Clontech) with limited (10 - 15) number of cycles. The double stranded cDNA was digested with *SfiI*, size fractionated through Sephacryl - 500 column (Amersham), and directionally cloned into the *SfiI *predigested vector DNR-LIB (Clontech). The first full-length cDNA library from liver (UAhib) was constructed without SMART template-switching and PCR amplification as described [39], and inserts were cloned into vector pCMV-SPORT6 (Invitrogen). Libraries were transformed to DH10B E. coli (Invitrogen) by using electroporation. Full details of the cDNA libraries constructed for this study are available at http://compbio.dfci.harvard.edu/tgi/cgi-bin/tgi/T_release.pl?gudb=bear. From each library, expressed sequence tags (EST) were generated from the 5'-end with the universal M13 forward primer.

### Genome Alignment of EST sequences and clustering

The genome sequence of 7.5X assembly of domestic dog was obtained from ENSEMBL (release 50, http://www.ensembl.org/Canis_familiaris/Info/Index). There are 23,550 genes annotated in the dog genome including the protein coding genes, pseudo gene, miRNA, rRNA, and tRNA. The bear ESTs were masked by RepeatMasker (-species mammal -norna) and then aligned on the dog genome by BLASTN with default parameters. The output file was parsed using E-value of 1.0 × 10^-10 ^as the upper limit and obtained the corresponding genomic sequences with 40 kb flanking region in both ends. We mapped the original ESTs on the extracted genomic sequences with SIM4 (A = 4, P = 1) to extract the alignment and splice site information. The ESTs alignment lengths on the genome longer than 50% of ESTs lengths and the alignment identity higher than 85% were used as the cutoff for assigning significant homology.

The EST alignments were clustered together with dog gene annotation (Canis_familiaris.BROADD2.50.gtf). We obtained "primary EST clusters", based on the same splicing sites and overlap, i.e., they were clustered if they shared at least one common splice site or they overlapped at least 50% length of the shorter sequence. Because of the duplication in the dog, there are redundancy existing in "primary EST Clusters", and we clustered them further if one cluster was a subset of the other and obtained "unique bear EST clusters".

### Bear tissue-specific gene expression

We carried out 2 × 2 fisher's exact test for fives tissues on 572 clusters which had at least 10 ESTs. P-value < 10^-3 ^and odds ratio > 10 were used as the criteria to identify the tissue specific genes. The expression profiles of five mammals (*Canis lupus familiaris*, *Homo sapiens*, *Mus musculus*, *Rattus norvegicus *and *Sus scrofa*) based on ESTs were downloaded from Unigene ftp http://www.ncbi.nlm.nih.gov/unigene. We compared 572 bear clusters with those of homologous genes in the other five species. For the genes with more than one Unigene clusters, the one with the largest EST copy number was selected. We obtained Pearson correlations and P-values under two-sided test between any two species.

### Alternative splicing analysis

In the bear EST clusters, un-spliced ESTs or spliced ESTs that had undetermined intron direction ("B" or "N" in the result of SIM4) or opposite orientation were discarded. Dog mRNA transcripts were filtered with same criteria. We only kept clusters with more than two transcripts which contained at least one bear EST. Splicing events were identified when two transcripts have at least one common splice site and one different splice site. An in-house script was used to identify the four types of alternative splicing pattern.

We applied the normalization method described in Eddo kim's paper, i.e., EST clusters with at least 10 unambiguously spliced ESTs were selected, then we randomly extracted 10 ESTs and performed alternative splicing analysis. We repeated the process for 100 times and the mean value of numbers of alternative splicing was calculated.

### Phylogenetic analysis

We selected the bear ESTs with identical splicing sites in the overlapping genomic region with the corresponding dog transcripts. CAP3 program was use to assemble the multiple ESTs of the same cluster into contig sequences [27]. The longest contig sequence was chosen to represents the longest bear transcript in each cluster. Orthologous gene information in mammalian species was retrieved from the ENSEMBL Compara database [40]. Protein coding sequences (CDS) of each orthologous gene were also retrieved from three high-quality genomes including human, cow and dog (ENSEMBL Version 50.1). Bear protein sequences were obtained by translating the bear transcripts using the same open reading frame as in other species.

The alignments of orthologous protein sequences from multiple species were done by the program MUSCLE using default parameters [41]. For each orthologous gene, we calculated non-synonymous rate (Ka) and synonymous rate (Ks) in pairwise species comparison using the codeml program of the PAML software package [42]. The group with more than one pair showing unusually high substitution rate (Ka > = 0.25 and/or Ks > = 5) was discarded. A super CDS was generated by concatenating the CDS fragments of aligned genes. For the super CDS alignment as well as each individual CDS alignment, the phylogenetic trees were generated by maximum likelihood methods based on different substitution models (GTR, TN93, T92, HKY85, F84, F81 and K80). Different substitution models yielded very similar results and GTR model with multiple hit corrections was eventually used in this study. Then, the Ka and Ks rates of each lineage were estimated with the codeml program under the free ratio model. For comparison, the Ka and Ks rates for the protein coding regions concatenated from 13 mitochondrial genes were also generated as the same method above.

### Likelihood ratio test for molecular evolution in bear

We removed the gaps and highly divergent regions showing contiguous mismatches in one lineage that could be caused by alternative splicing or frame-shift indels. The genes with the common length of multiple alignments less than 80% of the human CDS regions were also eliminated. The underlying phylogeny was assumed to be {{{bear, dog}, cow}, human} for all genes. The branch model in codeml of the PAML package was used to estimate the Ka/Ks ratio (ω) along the bear lineage and all other lineages. To test whether Ka/Ks ratio was significantly different between the bear lineage and other lineages, we computed the maximum likelihood ratio between two hypotheses: two different Ka/Ks ratios and constant Ka/Ks ratios along all lineages [42]. Two times the log likelihood ratio (2Δ) was transformed into p-values using chi-square test. P-value < 0.05 was used as the criterion to select candidate genes showing significant difference in Ka/Ks ratio. Further, branch-site model was implemented to identify the specific coding sites under positive selection. To increase the confidence of positive selection detection, we then later added homologous full-length coding regions available in other mammalian species including cat, microbat, megabat, alpaca, horse and hedgehog to the four species tree. The likelihood ratio test was based on the potential phylogeny {{{{{{bear, dog}, cat}, horse}, {microbat, megabat}}, {cow, alpaca}}, human} for *CSRP3 *and {{{{{dog, bear}, {microbat, megabat}}, cow}, hedgehog}, human} for *BPHL*.

### Database design

The web interface of the bear EST database was written in JavaServer Pages (JSP). After we mapped EST sequences to the dog genome, a track file containing the physical positions of each exon was created and then uploaded to UCSC web browser. The splicing patterns of all ESTs could be visualized under UCSC annotated platform.

## Abbreviations

EST: Expressed Sequence Tag; NJ: neighbor-joining; LRT: likelihood ratio test; MLP: muscle LIM protein; T-cap: titin-cap; CDS: coding sequences; BEB: Bayes empirical Bayes; *BPHL*: biphenyl hydrolase-like; *PLN: *Phospholamban; *CSRP3*: cysteine glycine-rich protein 3; *TNNI3: *Troponin I type 3; Ka: non-synonymous substitution rate; Ks: synonymous substitution rate.

## Authors' contributions

JY and VBF conceived the study. SZ and CS did the data analysis. AVG, NCS, and VBF constructed cDNA libraries and did EST sequencing. OT collected the bears used in this study. YX constructed on-line bear EST database. JY, CS, SZ, BMB, and VBF wrote the paper. All authors read and approved the final manuscript.

## Supplementary Material

Additional file 1 **Figure S1**. Distribution of bear EST lengths. Click here for file

Additional file 2 **Figure S2**. Distribution of sequence identities of bear EST to dog genome alignments. Click here for file

Additional file 3 **Figure S3**. Distribution of log2-transformed EST numbers per unique cluster. Click here for file

Additional file 4**Figure S4**. Phylogenetic trees of four mammalian species estimated by maximum likelihood method under GTR substitution model based upon the concatenated CDS regions from nuclear genes (A) and mitochondrial genes (B). Numbers below the branch are bootstrap supporting ratios and numbers above the branch are the average substitution numbers per site, which are related to Ka and Ks by  where Na and Ns are numbers of non-synonymous and synonymous sites.Click here for file

Additional file 5 **Table S1**. The complete list of 154 bear genes showing rapid evolution in bear lineage. Click here for file

Additional file 6 **Figure S5**. Multiple sequence alignments of full-length CSRP3 CDS nucleotide sequences in nine species. The LIM1 zinc-binding domain is marked with grey and the 60^th ^site is highlighted in blue. The corresponding protein sequence alignment is shown in Figure 5A. Click here for file
