# Supplementary figures and images for: Genomic analysis of expressed sequence tags in American black bear Ursus americanus
Source: BMC Genomics. 2010 Mar 26;11:201. doi: 10.1186/1471-2164-11-201 (PMC2996962; doi:10.1186/1471-2164-11-201)

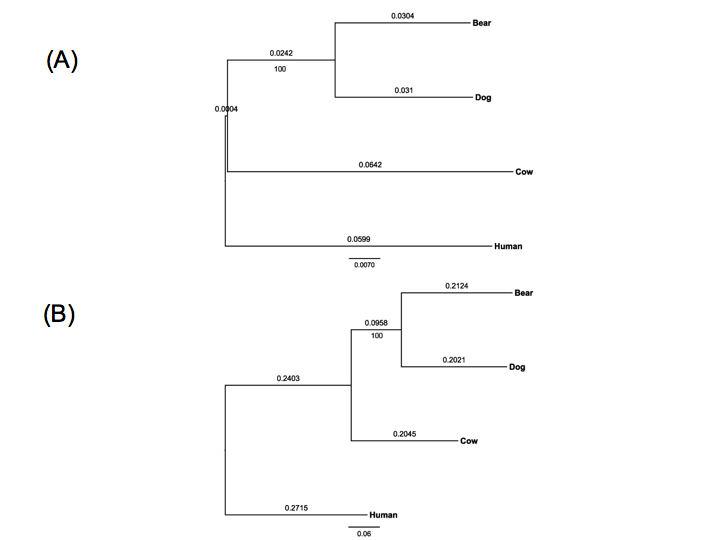

Supplement: Additional file 4 — Figure S4. Phylogenetic trees of four mammalian species estimated by maximum likelihood method under GTR substitution model based upon the concatenated CDS regions from nuclear genes (A) and mitochondrial genes (B). Numbers below the branch are bootstrap supporting ratios and numbers above the branch are the average substitution numbers per site, which are related to Ka and Ks by where Na and Ns are numbers of non-synonymous and synonymous sites. [file 1471-2164-11-201-S4.PNG]

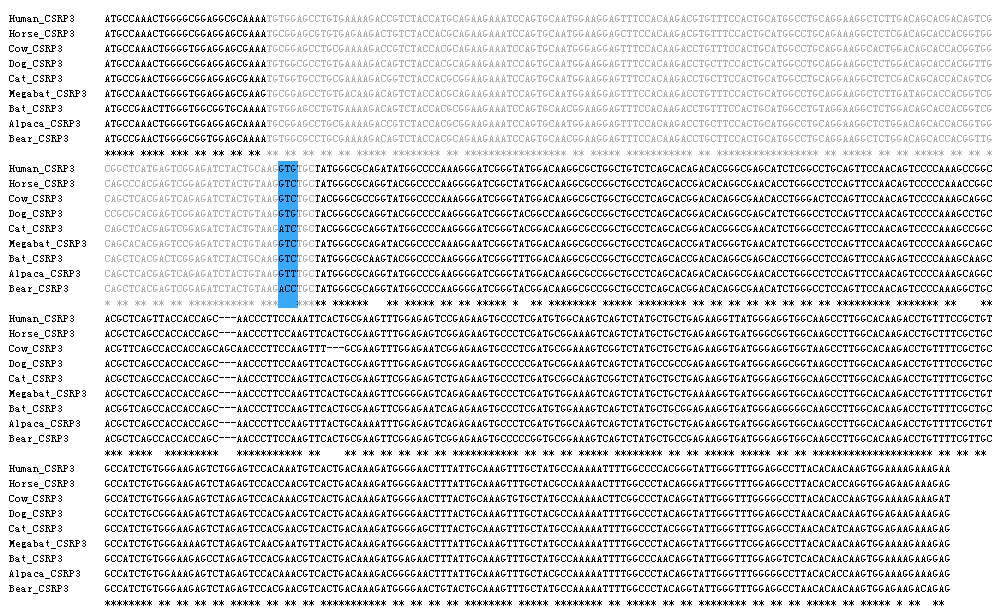

Supplement: Additional file 6 — Figure S5. Multiple sequence alignments of full-length CSRP3 CDS nucleotide sequences in nine species. The LIM1 zinc-binding domain is marked with grey and the 60th site is highlighted in blue. The corresponding protein sequence alignment is shown in Figure 5A. [file 1471-2164-11-201-S6.PNG]
